# Edible *Oxya chinensis sinuosa*—Derived Protein as a Potential Nutraceutical for Anticancer Immunity Improvement

**DOI:** 10.3390/nu12113236

**Published:** 2020-10-22

**Authors:** Woo Sik Kim, Jeong Moo Han, Ha-Yeon Song, Eui-Hong Byun, Ho Seong Seo, Eui-Baek Byun

**Affiliations:** 1 Advanced Radiation Technology Institute, Korea Atomic Energy Research Institute, Jeongeup 580-185, Korea; kws6144@kaeri.re.kr (W.S.K.); jmhahn@kaeri.re.kr (J.M.H.); songhy@kaeri.re.kr (H.-Y.S.); hoseongseo@kaeri.re.kr (H.S.S.); 2 Department of Biotechnology, College of Life Science and Biotechnology, Korea University, Seoul 136-701, Korea; 3 Department of Food Science and Technology, Kongju National University, Yesan 340-800, Korea; ehbyun80@kongju.ac.kr

**Keywords:** dendritic cells, *Oxya chinensis sinuosa*, protein, Th1, cytotoxic T cells, multifunctional T cells

## Abstract

*Oxya chinensis sinuosa* (Ocs) is consumed as representative edible insects in Asia, but its function in various immune systems remains unclear. This study aimed to demonstrate the immunomodulatory effect, particularly on the innate and adaptive immune response, of Ocs protein (Ocs-P) and to investigate its function as a potent anticancer immunostimulant when administered during the progression stage of colon carcinoma in tumor-bearing mice. Our in vitro results demonstrated that Ocs-P treatment induces phenotypic alteration (increased expression of surface molecules and production of T Helper type I-polarizing (Th1-polarizing) cytokines and decreased antigen uptake ability) of dendritic cells (DCs) through the activation of Mitogen-activated protein kinase (MAPK) and nuclear factor kappa B-dependent (NF-κB-dependent) signaling pathways. Additionally, Ocs-P-stimulated DCs initiated differentiation of naive T cells into IFN-γ-producing Th1-type T cells effectively and activated cytotoxic CD8^+^ T cell response. In colon carcinoma-bearing mouse models, oral administration of Ocs-P inhibited tumor growth and restored the expression of decreased surface molecules in lineage^-^CD11c^+^MHC-II^+^ splenic DCs. Furthermore, Ocs-P administration enhanced the generation of multifunctional CD4^+^ and CD8^+^ T cells expressing Th1-type cytokines (TNF-α, IFN-γ, and IL-2) and the degranulation marker (CD107a). Collectively, these results suggest that Ocs-P demonstrates an immunostimulatory effect and may induce powerful anticancer immunity.

## 1. Introduction

Insects have received attention for multiple purposes, not only as food and feed sources but also as living creatures that provide a variety of beneficial substances [1]. This considerable attention on insects originated from their advantages of being abundant and environmentally friendly; they constitute more than 80% of all living organisms on the earth and require less land and water in comparison with livestock [2,3,4]. Notably, insects have advantages regarding nutritional value; particularly, insects have high-quality proteins and are composed of 50%–70% total protein compared with plant and meat proteins [5,6,7,8]. Incidentally, insects have great potential to become one of the most promising protein sources, owing to their ability to solve the global problem of protein production regarding nutrition, environment, and economic value [7,9]. Apart from their nutritional roles, insect proteins have various pharmacological properties, such as antioxidant activity, anticancer activity, and a protective effect against atherosclerosis [10,11,12,13]. Water-soluble proteins derived from grasshoppers have strong antioxidant activities [14]. In addition, protein isolated from *Aspongopus chinensis* inhibits cancer cell proliferation by inducing apoptosis acceleration [15].

*Oxya chinensis sinuosa* (Ocs) is a grasshopper species belonging to the phylum Arthropoda (order, 54 Orthoptera; family, Acrididae; subfamily, Oxyinae), and the order Orthoptera contains the fourth most consumed edible insects, worldwide [1,14]. Traditionally in Korea, Ocs has been used as a remedy to cough, whooping cough, asthma, bronchitis, seizures, and paralysis [16]. Recently, the Korea Food & Drug Administration registered Ocs as a food in the Korean Food Standards Codex. Notably, Ocs proteins (Ocs-Ps) have been reported to enhance antioxidant, antimicrobial, and antiplatelet aggregation activities [10,14,17]. However, the immunological actions of Ocs-P, particularly on the innate and adaptive immune response, have not been fully investigated. A clearer immunological understanding of natural products may lead to the design of more successful nutraceuticals or functional materials; thus, there is an urgent need to better understand the immunological functions of Ocs-P. These studies could help provide a better understanding of its immunological effects on the use as edible insect.

Here, we investigated the immunomodulatory effect of Ocs-P in innate and adaptive immunity on the basis of dendritic cells (DCs), which are critical for initiating the adaptive immune response [18], and explored the major mechanisms controlling DC maturation. Additionally, immunological functions of Ocs-P as a potent immunostimulator that induces strong anticancer immunity were investigated.

## 2. Materials and Methods

### 2.1. Animal Studies

Bone marrow-derived dendritic cell (BMDC) differentiation and in vivo experiments were performed using 7- to 8-week-old C57BL/6 and BALB/c female mice from Orient Bio Inc. (Seoul, Korea). All animal studies were performed in accordance with the established guidelines of Korea Atomic Energy Research Institute (KAERI, Jeongeup, Korea) and approved by the Institutional Animal Care and Use Committee (IACUC) of KAERI (permit number: KAERI-IACUC-2019-001).

### 2.2. Antibodies and Reagents

For BMDC differentiation, primary cell culture was performed using recombinant mouse (rm)-granulocyte-macrophage colony-stimulating factor (rm-GM-CSF) and rm-interleukin-4 (rm-IL-4) from JW CreaGene (Daegu, Korea). As a positive control for in vitro BMDC maturation, ultrapure lipopolysaccharide (LPS) from *Escherichia coli* serotype 0111:B4 (Invivogen, San Diego, CA, USA) was used. For cytotoxic analysis, Annexin V and propidium iodide (PI) antibodies (Abs) were obtained from BD Bioscience (San Jose, CA, USA). The analysis of maturation, activation, and phenotypic alteration of DCs and T cells were performed via flow cytometry (FACSverse, BD Bioscience) using the following fluorescence-conjugated Abs: Live/Dead Cell Staining Kit (L/D; BV510) from Invitrogen (Carlsbad, CA, USA); Cell Proliferation Dye (CPD) eFluor 450, anti-major histocompatibility complex class I (-MHC-I; APC), -MHC-II (PE-Cy7), -cluster of differentiation 3 (CD3; APC-Cy7), -CD4 (Alexa488), -CD8α (Percp-Cy5.5), -IFN-γ (PE), -TNF-α (APC), and -IL-2 (PE-Cy7) Abs from eBioscience (San Diego, CA, USA); anti-CD11c (PE-Cy7), -CD80 (FITC), -CD86 (PE), -TNF-α (APC), -IL-12p70 (PE), -IL-10 (FITC), -CD107a (V450), -F4/80 (BV421), -B220 (BV421), -NK1.1 (BV421), -CD3e (BV421), and -Gr-1 (BV421) Abs from BD Bioscience. Analysis of isotype controls for DC surface molecule expression was performed using corresponding appropriate isotype Abs for surface molecules: rat IgG2 kappa (FITC), rat IgG2a kappa (PE), rat IgG2a kappa (APC), and rat IgG2b kappa (PE-Cy7) from BD Bioscience. Extracellular cytokine analysis was performed through enzyme-linked immunosorbent assay (ELISA) using the following mouse-specific ELISA kits: TNF-α, IL-12p70, IL-10, IFN-γ, IL-5, and IL-2 ELISA kits from BD Bioscience. Analysis of antigen (Ag) uptake ability was performed using FITC-conjugated 40,000 Da dextran (Sigma, St. Louis, MO, USA). Analysis of MAPK and NF-ĸB signals was performed using the following immunoblotting mouse Abs (mAbs) and signaling pathway-specific pharmacological inhibitors: phosphorylated (p)-extracellular-signal-regulated kinase (p-ERK), p-c-Jun N-terminal kinase (p-JNK), p-p38, p-inhibitor kappa B-alpha (p-IĸB-α), total-ERK, total-JNK, total-p38, IĸB-α, NF-ĸB, lamin B, and β-actin from Cell Signaling Technology (Boston, MA, USA); U0126 (ERK), SP600125 (JNK), SB203580 (p38), and Bay11-7082 (NF-ĸB) from Calbiochem (San Diego, CA, USA). Isolation of naive CD4^+^ and CD8^+^ T cells was performed with MACS MicroBeads system using CD4^+^ and CD8^+^ isolation kits (Miltenyi Biotec, San Diegom, CA, USA).

### 2.3. Isolation of Ocs-P Extract

Dried Ocs (100 g, Sworm Co., Seoul, Korea) was ground for 1 min using a manual grinder; the dried powder was mixed with 600 mL demineralized water containing 0.16% ascorbic acid, and this was subsequently incubated at 4 °C for 1 h. Following incubation, samples were ground again for 1 min, and the obtained solutions were centrifuged for 30 min at 70,000 rpm. Collected supernatants were filtered through Whatman No. 4 filter paper (Florham Park, NJ, USA) to remove the debris. Filtered solutions were dried in a vacuum freeze drier (VD-800F; Taitec, Saitama-ken, Japan), and freeze-dried powders (percentage yield of Ocs-P: 3.81%) were resuspended in phosphate-buffered saline (PBS, Biowest, Nuaille, France) and filtered through a 0.45 μm filter (Corning, NY, USA). Finally, we confirmed the absence of endotoxin or LPS contamination of purified Ocs-P using the Limulus Amebocyte Lysate (LAL) assay (Lonza, Basel Switzerland), following the manufacturer’s instructions.

### 2.4. Treatment Conditions of Ocs-P in BMDCs

Differentiation of BMDCs isolated from C57BL/6 mice were prepared as previously described [19]. Briefly, bone marrow cells were incubated for 8 d in the presence of RPMI-1640 medium (Biowest) supplemented with 10% heat-inactivated fetal bovine serum (FBS, Biowest), 1% penicillin/streptomycin (P/S, GIBCO, Carlsbad, CA, USA), 20 ng/mL GM-CSF, and 0.5 ng/mL IL-4. Thereafter, cells were harvested and conformed for BMDC purity (>90% purity) by FACSverse using anti-CD11c Ab. For Ocs-P treatment, BMDCs (1 × 10^6^/well in a 48-well plate) were stimulated with varying doses (1 to 100 μg/mL) of Ocs-P and incubated for 20 h at 37 °C/5% CO_2_. As a positive control for DC maturation, BMDCs were treated with 100 ng/mL LPS under Ocs-P-stimulated conditions.

### 2.5. Annexin V and PI Staining

Non-, LPS-, and Ocs-P-treated BMDCs (treated for 20 h) were harvested and stained with Annexin V and PI, according to the manufacturer’s protocol. Necrotic, late apoptotic, and apoptotic cell deaths were evaluated by detecting double or single positive cells of Annexin V and PI using FACSverse and FlowJo software (V10, BD Bioscience).

### 2.6. Analysis of Surface Molecules of BMDC

BMDCs (treated for 20 h) were stained with L/D, anti-CD80, -CD86, -MHC-I, and MHC-II Abs for 20 min at room temperature, washed with cold FACS washing buffer (2% FBS and 0.01% sodium azide in PBS), and finally fixed in IC fixation buffer (eBioscience). The surface expression levels of BMDCs were assessed using FACSverse and FlowJo software.

### 2.7. Detection of Intracelluar and Extracelluar Cytokines in BMDCs

For extracellular cytokine analysis, culture supernatants (treated for 20 h) of non-, LPS-, and Ocs-P-treated BMDCs were harvested, and TNF-α, IL-12p70, and IL-10 levels were measured with the respective ELISA kits, according to the manufacturers’ protocols. For intracellular cytokine analysis, BMDCs were treated with LPS and Ocs-P in the presence of GolgiPlug (BD Bioscience) for 8 h, and stimulated cells were subsequently collected and washed twice with cold FACS washing buffer. Thereafter, harvested cells were stained with anti-CD11c Ab for 20 min at room temperature and washed twice with cold FACS washing buffer. Cells were fixed and permeabilized using BD Cytofix/Cytoperm buffer for 20 min at room temperature and washed twice with BD Perm/Wash buffer. Following washing, cells were stained with anti-TNF-α, -IL-12p70, and -IL-10 Abs. Finally, cells were washed twice with BD Perm/Wash buffer, and intracellular cytokine levels in BMDCs (CD11c^+^ cells) were assessed using FACSverse and FlowJo software.

### 2.8. Analysis of Ag Uptake Ability in BMDCs

Non-, LPS-, and Ocs-P-treated BMDCs (treated for 20 h) were incubated with 1 mg/mL dextran for 40 min at 37 °C and 4 °C. Cells were washed three times with cold FACS washing buffer and stained with anti-CD11c Ab for 30 min at 4 °C. Thereafter, cells were washed and fixed with IC fixation buffer, and the uptake levels (CD11c^+^Dextran^+^ cells) of dextran in BMDCs were assessed using FACSverse and FlowJo software.

### 2.9. Western Blotting Analysis

Cytosolic and nuclear proteins were isolated by cell lysis with the Pierce RIPA buffer (Rockford, IL, USA) and the CelLytic NuCLEAR Extraction Kit (Sigma), respectively. The subsequent steps were performed as described previously [20].

### 2.10. Ocs-P-treated BMDC Maturation Induced by Inhibition of the MAPK and NF-ĸB Signaling Pathways

BMDCs were pretreated with each MAPK (U0126; 10 μM, SP600125; 20 μM, SB20358; 20 μM), and NF-ĸB (Bay11-7082; 10 μM) signaling inhibitor for 2 h prior to Ocs-P stimulation for 20 h. Thereafter, the ELISA was used to measure extracellular cytokine levels (TNF-α and IL-12p70) in the cell culture supernatants. Additionally, cells were analyzed for surface molecules (CD80, CD86, MHC-I and MHC-II) of DCs using FACSverse.

### 2.11. Allogenic Mixed Lymphocyte Reaction

Splenic CD4^+^ and CD8^+^ T cells from BALB/c mice were isolated using MACS CD4^+^ and CD8^+^ isolation kits. Isolated T cells were incubated with 1 μM Cell Proliferation Dye (CPD) eFluor 450 in a 37 °C water bath under dark conditions. Following 15 min of staining, cells were washed three times with cold PBS containing 10% FBS. Thereafter, CPD-labeled T cells (5 × 10^5^/well in a 96-well plate) were co-cultured with non-, LPS-, and Ocs-P-treated BMDCs (1 × 10^5^/well in a 96-well plate) in the presence of RPMI-1640 medium containing 10% FBS and 1% P/S. Following 3 d of culturing, extracellular cytokine levels (IFN-γ, IL-5, and IL-2) were measured in the cell culture supernatants using ELISA. Thereafter, each CD4^+^ and CD8^+^ T cell was harvested and washed twice with cold FACS washing buffer. Harvested CD4^+^ and CD8^+^ T cells were subsequently stained with anti-CD4 and -CD8 Abs, respectively. Finally, cells were washed twice with cold FACS washing buffer and fixed with IC fixation buffer, and proliferation levels (percentage of CPD-negative T cells) of CD4^+^ and CD8^+^ T cells were assessed using FACSverse and FlowJo software.

### 2.12. Cytotoxic T Lymphocyte (CTL) Function Assay

BMDCs were stimulated with Ocs-P (100 μg/mL) in the absence and presence of 1 μg/mL Ovalbumin_257-264_ (OVA_257-264_) peptide (SIINFEKL, AbFrontier, Seoul, Korea) bound to H-2Kb of MHC-I. Following a 20 h treatment, cells were harvested and washed twice with cold PBS. Thereafter, C57BL/6 mice were immunized intravenously three times (on days 1, 3, and 5) with PBS (PBS-injected group) or each DC (2 × 10^6^/mouse; non-treated DC, OVA_257-264_-treated DC, and DC treated with OVA_257-264_ and Ocs-P). After 5 d of the last immunization, naive splenic T cells were prepared from C57BL/6 mice, incubated in the presence and absence of OVA_257-264_ peptide for 2 h, harvested, and washed twice with cold PBS. Among these samples, OVA_257-264_-treated T cells were stained with a high-dose Cell Proliferation Dye (CPD, 5 mM) in a 37 °C water bath under dark conditions for 15 min. In contrast, non-treated T cells were stained with low-dose CPD (0.5 mM). Thereafter, CPD^high^-stained OVA_257-264_-treated T cells (5 × 10^6^/mouse) and CPD^low^-stained non-treated T cells (5 × 10^6^/mouse) were mixed together at 1:1 ratio and were intravenously injected into PBS- and DC-immunized C57BL/6 mice. After 6 h, mice were sacrificed, and spleen cells were isolated from each group. Finally, cytotoxic activity via decreased levels of CPD^high^-stained T cells from each spleen cell was assessed using FACSverse and FlowJo software.

### 2.13. Animal Experiment for Antitumor Effect

The mouse colon carcinoma cell line CT26 (purchased from the American Type Culture Collection) used in in vivo experiments was maintained at 37 °C/5% CO_2_ in the presence of RPMI-1640 medium containing 10% FBS and 1% P/S; six passages of cells were performed for in vivo antitumor experiments. These cells (1 × 10^5^/mouse) were subsequently washed and inoculated subcutaneously into naive BALB/c mice. At 6 d after tumor inoculation, mice were orally administered with Ocs-P (10 mg/mouse) five times at 3 d intervals, and tumor size was calculated seven times every 3 d using the formula: area = (length × wideth^2^)/2.

For analysis of splenic DC maturation induced by Ocs-P administration, 24 d after tumor inoculation, mice were sacrificed, and single-cell suspensions of spleens were isolated. These were stained with L/D, lineage cocktail (anti-Gr-1 Ab; neutrophil marker, anti-F4/80 Ab; macrophage marker, anti-B220 Ab; B cell marker, anti-NK1.1 Ab; NK cell marker, and anti-CD3e Ab; T cell marker), anti-CD11c, -CD80, -CD86, -MHC-I, and -MHC-II Abs for 30 min at 4 °C. Cells were washed twice with cold FACS washing buffer and then fixed. Expression levels of surface molecules in splenic DCs (L/D^-^lineage^-^CD11c^+^MHC-II^+^) were evaluated using FACSverse and FlowJo software.

For analysis of multifunctional CD4^+^ and CD8^+^ T cell responses induced by Ocs-P administration, 24 d after tumor inoculation, mice were sacrificed, and splenic T cells were isolated. Splenic T cells (1 × 10^6^/mL) were stimulated with 1× eBioscience Cell Stimulation Cocktail (plus protein transport inhibitor) in the presence of anti-CD107a Ab for 4 h at 37 °C/5% CO_2_. Stimulated cells were washed twice with cold FACS washing buffer and stained with L/D, anti-CD3, -CD4, and -CD8 Abs for 30 min at 4 °C. Thereafter, cells were washed twice with cold FACS washing buffer, fixed and permeabilized using BD Cytofix/Cytoperm buffer for 20 min at room temperature, and washed twice with BD Perm/Wash buffer. Thereafter, cells were stained anti-IFN-γ, -IL-2, and -TNF-α Abs. Finally, cells were washed twice with BD Perm/Wash buffer, and intracellular cytokine and CD107a levels in CD4^+^ (L/D^-^CD3^+^CD4^+^) and CD8^+^ (L/D^-^CD3^+^CD8^+^) T cells were assessed using FACSverse and FlowJo software.

### 2.14. Statistical Analysis

Data were assessed using Tukey’s unpaired t-tests or multiple comparison tests with GraphPad Prism 7 (2018, GraphPad, San Diego, CA, USA). Data are represented as the mean ± standard deviation (SD). * *p* < 0.05, ** *p* < 0.01, and *** *p* < 0.001 were considered statistically significant.

## 3. Results

### 3.1. Ocs-P Promotes a Typical Maturation Profile for Potential Th1 Polarization and Cytotoxic CD8^+^ T Cell Responses in Primary BMDCs

**Figure 1:**
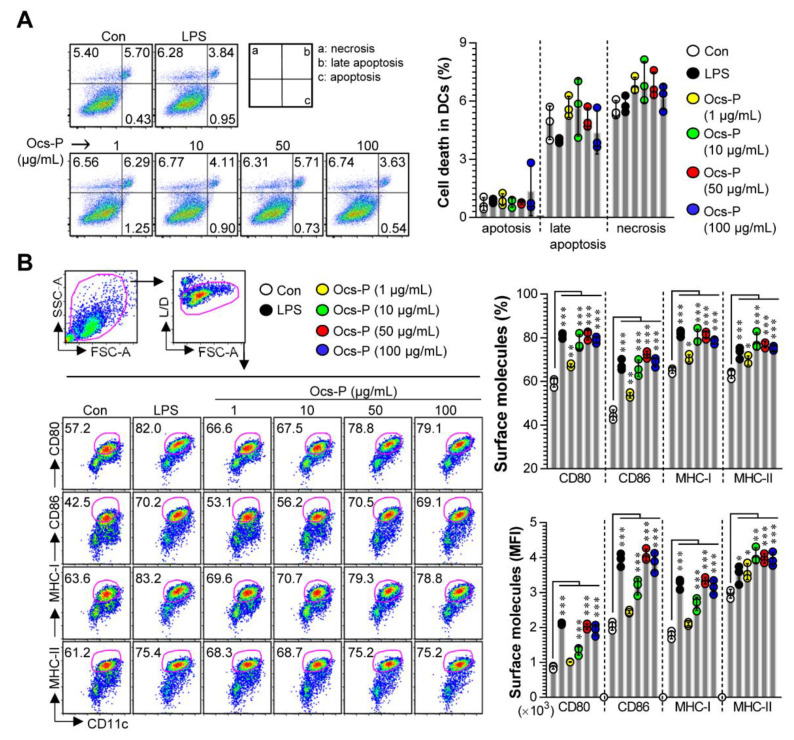
Cytotoxicity and phenotype characteristics induced by Ocs-P treatment in in vitro cultured BMDCs. (**A**) Immature BMDCs were subjected to LPS (100 ng/mL) or Ocs-P (1 to 100 μg/mL) treatment for 20 h or no treatment (Con), and dead cells (necrotic, late apoptotic, apoptotic cell death) were measured by Annexin V and PI staining; (**B**) Non- (Con), LPS-, and Ocs-P-treated DCs were stained with surface antibodies (anti-cluster of differentiation 80 (anti-CD80), -CD86, -major histocompatibility complex class I (-MHC-I), and -MHC-II) and pan-DC marker (anti-CD11c), and the expression levels (percentage and mean fluorescence intensity; MFI) of co-stimulatory (CD80 and CD86) and MHC class molecules (MHC-I and MHC-II) in CD11c^+^ cells were analyzed using flow cytometry. All data (dot plot and bar graphs) are representative of three independent experiments with similar results. Data represent the mean ± SD (three samples for all experimental conditions); * *p* < 0.05, ** *p* < 0.01, or *** *p* < 0.001. SD; standard deviation.

Th1 and CTL responses play a major role in protecting and eliminating tumor progression [21]. Notably, activation, proliferation, and differentiation of these T cells require the Th1-polarizing cytokines (TNF-α and IL-12p70) predominantly produced by Ag presenting cells (APCs; macrophages and DCs) and the interactions of APCs and T cells by upregulating surface molecules (CD80, CD86, MHC-I, and MHC-II) on APCs [22,23,24,25]. Thus, we confirmed whether Ocs-P could induce DC maturation with production of Th1-polarizing cytokines and upregulation of surface molecules. Prior to this, the cytotoxic effect of Ocs-P in various concentrations was described in BMDCs, as cytotoxicity can affect DC maturation. Therefore, ≤100 μg/mL Ocs-P treatment of BMDCs demonstrated no significant increase in Annexin V^+^ and/or PI^+^ cells (Figure 1A). Thus, in vitro experiments for DC maturation were conducted. Thereafter, we analyzed the expression of various surface molecules in LPS (positive control for DC maturation)- and Ocs-P (1 to 100 μg/mL)-treated BMDCs. As shown in Figure 1B, expression levels (percentage and MFI value) of CD80, CD86, MHC-I, and MHC-II of BMDCs significantly increased in a dose-dependent manner by Ocs-P treatment.

**Figure 2:**
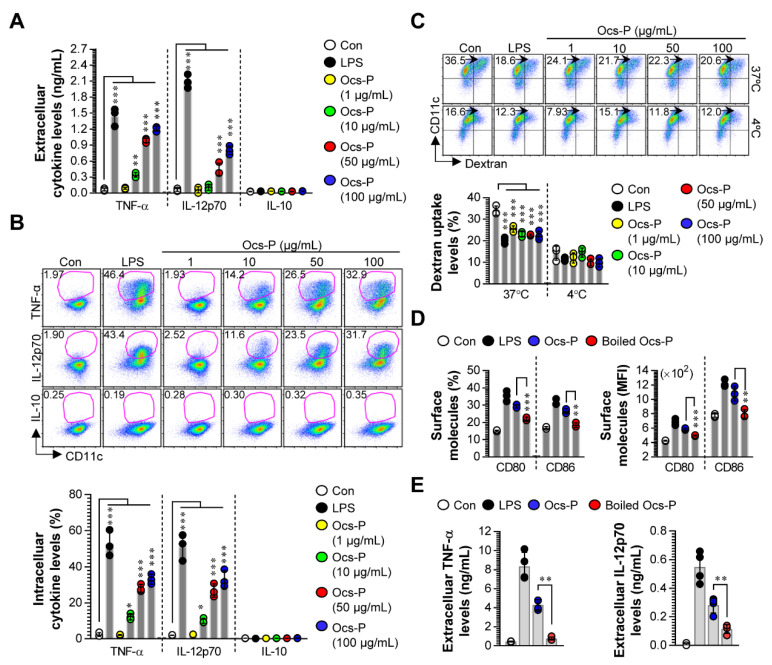
Analysis of cytokine profiles, antigen uptake ability, and surface molecules induced by Ocs-P or boiled Ocs-P treatment in in vitro cultured BMDCs. (**A**) Immature BMDCs were subjected to LPS (100 ng/mL) or Ocs-P (1 to 100 μg/mL) for 20 h or no treatment (Con), and culture supernatants were harvested. Extracellular cytokine levels (TNF-α, IL-12p70, IL-10) were analyzed using ELISA; (**B**) DCs were treated with LPS and Ocs-P in the presence of GolgiPlug for 8 h and stained with pan-DC marker (anti-CD11c) and intracellular cytokine detection antibodies (anti-TNF-α, -IL-12p70, and -IL-10). Intracellular cytokine levels were detected using flow cytometry; (**C**) Non- (Con), LPS-, and Ocs-P-treated DCs (treated for 20 h) were incubated with FITC-dextran at 37 °C and 4 °C for 40 min and stained with anti-CD11c Ab. Dextran uptake ability was measured using flow cytometry; (**D**,**E**) BMDCs were treated with LPS, Ocs-P (100 μg/mL), and boiled Ocs-P (100 μg/mL) for 20 h. (**D**) Cells were stained with anti-CD11c, -CD80, and -CD86 Abs, and the expression levels of CD80 and CD86 in CD11c^+^ cells were analyzed (**E**) Extracellular cytokine levels were analyzed in each culture supernatant using ELISA. All data (dot plots and bar graphs) are representative of three independent experiments with similar results. Data represent the mean ± SD (three samples for all experimental conditions); * *p* < 0.05, ** *p* < 0.01, or *** *p* < 0.001. SD; standard deviation.

Under the experimental condition illustrated in Figure 1, Ocs-P-treated BMDCs significantly increased the production of extracellular TNF-α and IL-12p70; however, BMDCs did not increase IL-10 levels, a potent anti-inflammatory immunosuppressive cytokine (Figure 2A). These results were confirmed by the analysis of intracellular cytokine levels using flow cytometry (Figure 2B). Furthermore, immature DCs show high ability for Ag uptake, whereas mature DCs lose their ability to take up Ags [26], indicating that the analysis of Ag uptake ability is a critical step to confirm DC maturation. In this study, we hypothesized that Ocs-P treatment can induce decreased Ag uptake ability in BMDCs. The functional change for Ag uptake induced by Ocs-P treatment in BMDCs was analyzed using FITC-labeled dextran. As expected, LPS (positive control)- and Ocs-P-treated BMDCs displayed significantly decreased cellular uptake levels of dextran (Figure 2C). Importantly, the expression of surface molecules and immunostimulatory cytokines (TNF-α and IL-12p70) in 1 μg/mL Ocs-P-treated DCs was similar or slightly higher than non-treated DCs, while dextran uptake levels was significantly reduced. These results indicate that 1 μg/mL Ocs-P also have potential to induce the maturation of DCs. However, to investigate the accurate assessment for Ocs-P-induced DC maturation, concentration of 100 μg/mL of Ocs-P was selected for further investigation. Thereafter, to confirm that Ocs-P-induced DC maturation is not caused by contamination of other soluble factors, protein (Ocs-P) denaturation was induced by heating at 100 °C for 30 min, and cells were subsequently treated. Therefore, heat denaturation of Ocs-P abrogated the ability of Ocs-P-induced DC maturation (Figure 2D; expression of surface molecules, Figure 2E; production of Th1 cytokines). We further analyzed the amount of endotoxin in the Ocs-P using the LAL assay. However, endotoxin content of Ocs-P was <4 pg/mL (<0.1 EU/mL), which indicated that maturation of DCs induced by Ocs-P was not due to contaminating endotoxins or LPS. These results, together with the potent ability to induce secretion of predominant Th1-polarizing cytokines and the expression of surface molecules, indicate that Ocs-P can induce phenotypic and functional maturation of DCs that generate Th1 and CTL responses.

### 3.2. Ocs-P Induces DC Maturation by Activating MAPK and NF-ĸB Signals

**Figure 3:**
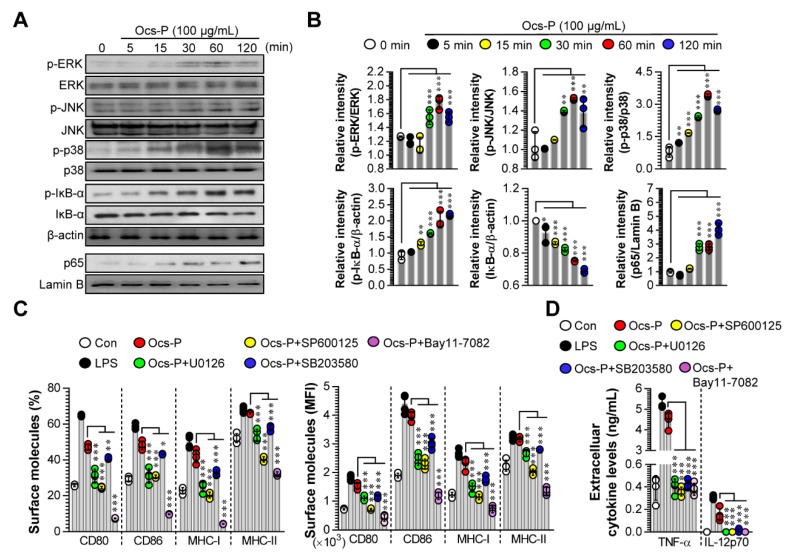
Effect of MAPK and NF-ĸB signals in Ocs-P-treated DC maturation. (**A**) BMDCs were stimulated with Ocs-P for 0 (non-treated DCs), 5, 15, 30, and 60 min, and activation of Mitogen-activated protein kinase (MAPK) (phosphorylated extracellular-signal-regulated kinase (ERK), c-Jun N-terminal kinase (JNK), p38) and NF-ĸB (phosphorylated inhibitor kappa B-alpha (IĸB-α), IĸB-α degradation, nuclear translocation of p65) signals were analyzed via western blotting; (**B**) The relative intensity for MAPK and NF-ĸB signals were normalized with β-actin or lamin B; (**C**,**D**) Cells were pretreated with inhibitors of each MAPK (ERK; U0126, JNK; SP600125, p38; SB203580) and NF-ĸB (Bay11-7082) signal for 2 h and subsequently stimulated with Ocs-P for 20 h. (**C**) Analysis of surface molecules using flow cytometry. (**D**) Analysis of extracellular cytokines using ELISA. All data (western blot data and bar graphs) are representative of three independent experiments with similar results. Data represent the mean ± SD (three samples for all experimental conditions); * *p* < 0.05, ** *p* < 0.01, or *** *p* < 0.001. SD; standard deviation.

We investigated the signaling molecular mechanism for DC maturation induced by Ocs-P treatment. We focused on the MAPK and NF-ĸB signaling pathways, as activation of these signals are important for inducing DC maturation [27]. Thus, we hypothesized that Ocs-P-induced DC maturation requires activation of MAPK and NF-ĸB signals. These signals were measured through western blotting after Ocs-P treatment at various time points. Notably, Ocs-P could activate MAPK and NF-ĸB signals by stimulating MAPK (ERK, JNK, p38) phosphorylation, IĸB-α phosphorylation/degradation, and p65 nuclear translocation in DCs (Figure 3A,B). Thereafter, we analyzed whether the expression of surface molecules and production of Th1-polarizing cytokines induced by Ocs-P require MAPK and NF-ĸB signals. These experiments were performed in the presence of MAPK and NF-ĸB inhibitors. In Ocs-P-induced DC maturation, inhibition of MAPK and NF-ĸB signals showed significantly reduced surface molecule expression (Figure 3C; CD80, CD86, MHC-I, MHC-II) and Th1-polarizing cytokines (Figure 3D; TNF-α, IL-12p70). These results indicate that MAPK and NF-ĸB signals are involved in Ocs-P-induced DC maturation.

### 3.3. Ocs-P-stimulated DCs Induce T Cell Proliferation, Favor Th1 Polarization, and Activate Cytotoxic CD8^+^ T Cells

**Figure 4:**
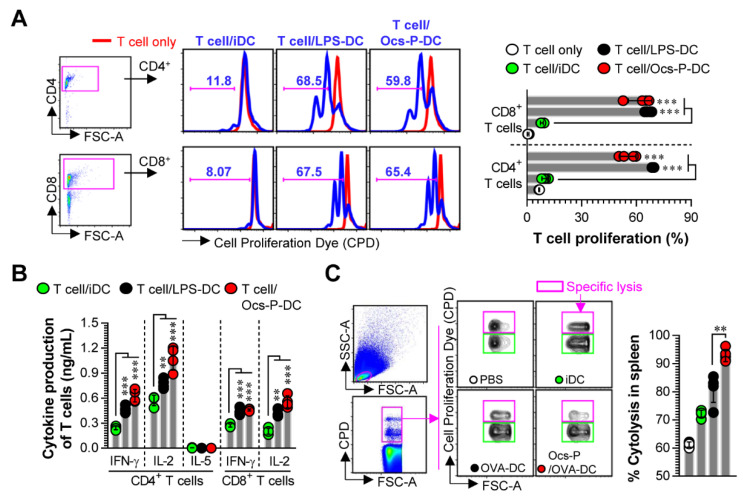
Analysis of proliferation, activation, and differentiation of T cells induced by Ocs-P-treated DCs. (**A**,**B**) Analysis of proliferation and differentiation of T cells induced by Ocs-P-treated DCs was measured using the allogenic MLR assay as described in the *Materials and Methods*. (**A**) Lymphocyte division using Cell Proliferation Dye (CPD)-stained CD4^+^ and CD8^+^ T cells co-cultured with each DC was analyzed using flow cytometry; (**B**) Type and activation (Th1: IFN-γ, IL-2 secretion; Th2: IL-5 secretion; activated CD8^+^ T cells: IFN-γ, IL-2 secretion) of T cells were evaluated in culture supernatants of T cells co-cultured with each DC. Histogram data and bar graphs are representative of three independent experiments with similar results. Data represent the mean ± SD (four samples for all experimental conditions). T cell/iDC; CD4^+^ or CD8^+^ T cells co-cultured with non-treated DCs, T cell/LPS-DCs; CD4^+^ or CD8^+^ T cells co-cultured with LPS-treated DCs, T cell/Ocs-P-DCs; CD4^+^ or CD8^+^ T cells co-cultured with Ocs-P-treated DCs. (**C**) CTL activity was measured by OVA-specific killing effect induced by the injection of CPD^low^- and CPD^high^-labeled splenocytes in mice immunized with non-treated DCs (iDC)-, OVA_257-264_-treated DCs (OVA-DC), and DCs (Ocs-P/OVA_257-264_-DC) treated with Ocs-P and OVA_257-264_. Data are representative of two independent experiments with similar results. Data represent the mean ± SD (five mice per group); ** *p* < 0.01, or *** *p* < 0.001. SD; standard deviation.

Having established that Ocs-P induces DC maturation by MAPK and NF-ĸB activation, we evaluated whether Ocs-P-treated DCs can induce anticancer T cell immunity, such as Th1-type T cell immunity and CTL response. As shown in Figure 4A,B, CD4^+^ and CD8^+^ T cells co-cultured with LPS- and Ocs-P-treated DCs proliferated to a considerably greater extent (Figure 4A) and produced more Th1 cytokines (Figure 4B; IFN-γ, IL-2) than T cells co-cultured with non-treated DC (iDC), while IL-5 (Figure 4B; Th2 cytokine) levels showed no difference. Therefore, we subsequently analyzed in vivo CTL activity for Ocs-P-treated DCs (Figure 4C). To accomplish this, mice from each group were immunized with PBS, iDC, OVA_257-264_-treated DCs, and Ocs-P/OVA_257-264_-treated DCs. Five days after final immunization, mice were injected with mixed T cells (1:1 ratio; Cell Proliferation Dye^high^-stained OVA_257-264_-treated T cells and Cell Proliferation Dye^low^-stained non-treated T cells). For OVA-specific CTL response, Ocs-P/OVA_257-264_-treated DC-immunized groups showed a significantly reduced percentage (this phenomenon means induction of CTL activity) of Cell Proliferation Dye^high^-stained OVA_257-264_-treated T cells compared with OVA_257-264_-treated DC-immunized groups. These findings imply a strong possibility that Ocs-P could induce strong anticancer T cell immune responses.

### 3.4. Ocs-P Inhibits Tumor Growth via Activation of Innate and Adaptive Immunity

**Figure 5:**
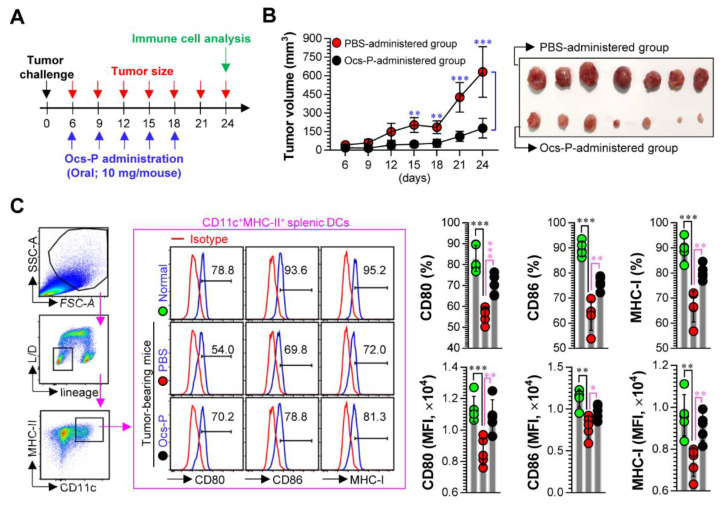
Analysis of protective efficiency and DC phenotypic alteration induced by Ocs-P administration in tumor-bearing mice. (**A**) Protocol for in vivo experiment (the effect of Ocs-P administration in CT26 tumor-bearing mouse model); (**B**, left panel) Analysis of tumor growth at indicated time points after tumor challenge; (**B**, right panel) Photos of tumors induced by PBS and Ocs-P administration in CT26 tumor-bearing mice. Tumor size data represent the mean ± SD (seven mice per group); (**C**) Expression of surface molecules (CD80, CD86, and MHC-I) in lineage^-^CD11c^+^MHC-II^+^ splenic DCs from normal mice, PBS-administered tumor-bearing mice, and Ocs-P-administered tumor-bearing mice. All data are representative of two independent experiments with similar results. Bar graphs represent the mean ± SD (five mice per group); * *p* < 0.05, ** *p* < 0.01, or *** *p* < 0.001. SD; standard deviation. *n. s.*; not significant.

Based on the above results, we hypothesize three prediction results: (i) inhibitory effect of tumor growth by Ocs-P administration in tumor-bearing mice, (ii) DC maturation by Ocs-P administration during tumor progression, and (iii) induction of strong anticancer T cell immunity by Ocs-P administration in tumor-bearing mice. To explore this hypothesis, an animal study for anticancer effect and immunological contribution induced by Ocs-P administration was performed (Figure 5A). As expected, in the colon carcinoma-bearing mouse model, oral administration of Ocs-P exhibited significantly reduced tumor growth compared with PBS administration (Figure 5B). When mature phenotypes of lineage^-^CD11c^+^MHC-II^+^ splenic DCs in the spleens isolated from each group (normal group, PBS-administered tumor-bearing group, Ocs-P-administered tumor-bearing group) were assessed, PBS-administered tumor-bearing mice showed lower expression levels of surface molecules (CD80, CD86, MHC-I) in lineage^-^CD11c^+^MHC-II^+^ splenic DCs compared with normal mice. Notably, Ocs-P administration restored the expression of CD80, CD86, and MHC-I in lineage^-^CD11c^+^MHC-II^+^ splenic DCs induced by tumor injection (Figure 5C).

**Figure 6:**
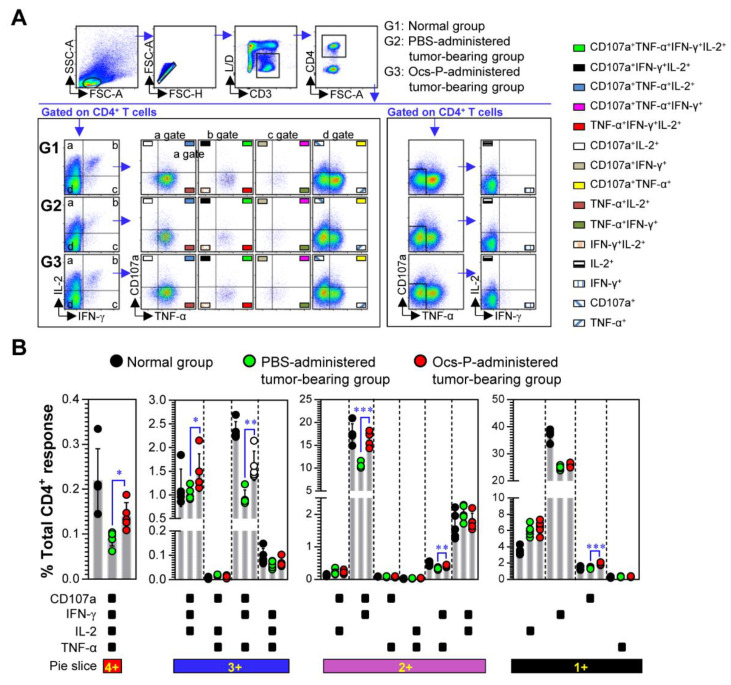
Analysis of multifunctional CD4^+^ T cell responses induced by Ocs-P administration in tumor-bearing mice. At 24 d after tumor challenge, single-cell suspensions of the spleens from each group (normal group, PBS-administered tumor-bearing group, and Ocs-P-administered tumor-bearing group) were isolated and treated with Cell Stimulation Cocktail (plus protein transport inhibitor) in the presence of anti-CD107a Ab for 4 h. Thereafter, cells were stained with L/D, anti-CD3, -CD4, -IFN-γ, -IL-2, and -TNF-α Abs as described in the *Materials and Methods*. (**A**) Gating strategy for multifunctional CD4^+^ T cells via flow cytometry analysis. Forward scatter area; FSC-A, forward scatter height; FSC-H, side scatter height; SSC-A; (**B**) The percentage of single, double, triple, or quadruple functional CD4^+^ T cells in three groups are indicated in bar graphs. All data are representative of two independent experiments with similar results. Bar graphs represent the mean ± SD (five mice per group); * *p* < 0.05, ** *p* < 0.01, or *** *p* < 0.001. SD; standard deviation.

**Figure 7:**
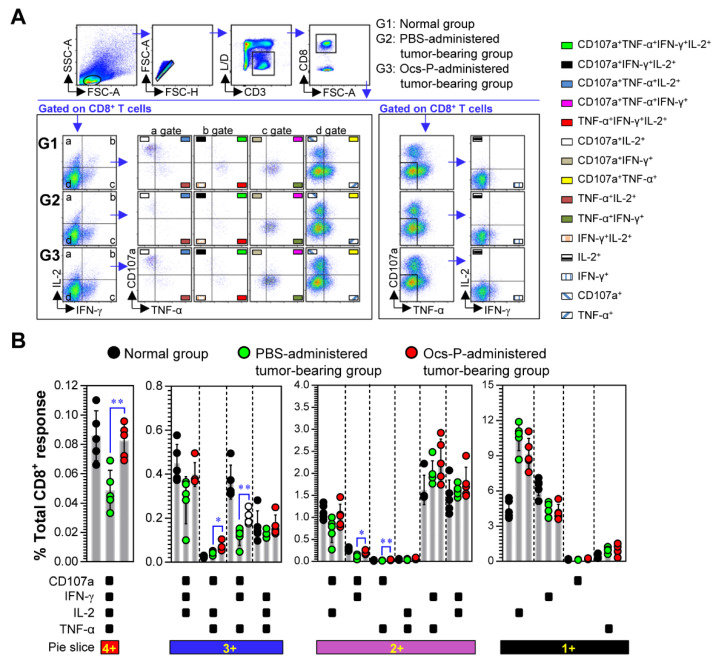
Analysis of multifunctional CD8^+^ T cell responses induced by Ocs-P administration in tumor-bearing mice. At 24 d after tumor challenge, single-cell suspensions of the spleens from each group (normal group, PBS-administered tumor-bearing group and Ocs-P-administered tumor-bearing group) were isolated, and treated with Cell Stimulation Cocktail (plus protein transport inhibitor) in the presence of anti-CD107a Ab for 4 h. Thereafter, cells were stained with L/D, anti-CD3, -CD8, -IFN-γ, -IL-2, and -TNF-α Abs as described in the *Materials and Methods*. (**A**) Gating strategy for multifunctional CD8^+^ T cells by flow cytometry analysis; (**B**) The percentage of single, double, triple, or quadruple functional CD8^+^ T cells in three groups are indicated in bar graphs. All data are representative of two independent experiments with similar results. Bar graphs represent the mean ± SD (five mice per group); * *p* < 0.05, ** *p* < 0.01. SD; standard deviation.

Furthermore, we confirmed that Ocs-P induced T cell immunity in tumor-bearing mice. Notably, multifunctional CD4^+^ and CD8^+^ T cells capable of producing multiple Th1 cytokines (IFN-γ, TNF-α, IL-2) and expressing the degranulation marker (CD107a) play a critical role in cancer protection [28,29]. Thus, we further focused the functional phenotype for CD4^+^ and CD8^+^ T cell immune responses between the two groups (PBS-administered and Ocs-P-administered tumor-bearing group). To accomplish this, spleen cells from each group were evaluated for the frequency of the single, double, triple, or quadruple functional CD4^+^ (Figure 6A) and CD8^+^ T cells (Figure 7A) by flow cytometry using intracellular staining for multiple cytokines. As a result, CD4^+^ T cell response induced by oral administration of Ocs-P displays a high degree of multifunctionality with increased CD107a^+^IFN-γ^+^IL-2^+^TNF-α^+^ (up to 1.5-fold), CD107a^+^IFN-γ^+^IL-2^+^ (up to 1.4-fold)-, CD107a^+^IFN-γ^+^TNF-α^+^ (up to 1.7-fold)-, CD107a^+^IFN-γ^+^ (up to 1.6-fold)-, and IFN-γ^+^TNF-α^+^ (up to 1.3-fold)-producing T cells compared with the PBS-administered tumor-bearing group (Figure 6B).

Thereafter, when CD8^+^ T cell responses were analyzed, Ocs-P-administered tumor-bearing group showed higher frequencies of CD107a^+^IFN-γ^+^IL-2^+^TNF-α^+^ (up to 1.7-fold), CD107a^+^IL-2^+^TNF-α^+^ (up to 1.6-fold)-, CD107a^+^IFN-γ^+^TNF-α^+^ (up to 1.7-fold)-, CD107a^+^IFN-γ^+^ (up to 1.8-fold)-, and CD107a^+^TNF-α^+^ (up to 1.8-fold)-producing T cells compared with the PBS-administered tumor-bearing group (Figure 7B). Collectively, our results suggest that Ocs-P could improve DC maturation inhibited by cancer progression, and this DC maturation may contribute to the upregulation of multifunctional CD4^+^ and CD8^+^ T cells.

## 4. Discussion

This study aimed to investigate the immunological contribution of Ocs-P in DCs and showed that Ocs-P functionally converts immature DCs into immunogenic mature DCs with functional and phenotypic change that increases the production of Th1-polarizing cytokines and the expression of surface molecules by MAPK- and NF-ĸB-dependent signaling pathways. Additionally, these Ocs-P-stimulated mature DCs promoted proliferation and activation of naive CD4^+^ and CD8^+^ T cells along with increased IFN-γ and IL-2 production, suggesting the potential of Ocs-P in providing anticancer immunity.

In fact, several studies have suggested that successful therapy in the initiation, promotion, and progression stages of tumors rely on the ability of DCs, as effective and reliable DC maturation can initiate antitumor T cell immunity, including Th1 and activated CD8^+^ T cell responses (or CTL response) [30,31,32]. However, in tumor microenvironments, tumor-mediated soluble factors, such as cytokines (IL-10, IL-6, TGF-β), enzymes (indoleamine 2,3-dioxygenase, arginase), and lipid mediators (prostaglandins), impair the phenotypic and functional properties of DCs and skew their differentiation by bone marrow precursors toward immunosuppressive and tolerogenic DCs (or regulatory DCs) [33,34]. Eventually, these cells inhibit anticancer T cell immunity and thus stimulate tumor development and growth [35]. Therefore, to effectively control tumors, it is necessary to restore immunosuppressive DC function or eliminate tumor-mediated soluble factors.

Recently, phenotypes and properties of tumor-associated DCs have been extensively studied. DCs isolated from several tumor models and cancer patients were found to possess tolerogenic properties, such as low levels of co-stimulatory molecules (CD80, CD86) and low production of Th1-polarizing cytokine IL-12 [18,36,37]. Notably, we observed similar results in our in vivo experiment (Figure 5C). The expression levels of CD80, CD86, and MHC-I on CD11c^+^MHC-II^+^ splenic DCs from mice inoculated with murine colon carcinoma cells were lower when compared with normal mice. Notably, Ocs-P administration has been shown to inhibit tumor growth and restore expression of decreased surface molecules in CD11c^+^MHC-II^+^ splenic DCs of colon carcinoma-bearing mice. Here, focused DCs are an extremely promising cell type for cancer immunotherapy [38]. Thus, while the functional role of DCs induced by Ocs-P administration for differentiation and activation of T cells requires more directed study, our results predicted that Ocs-P-mediated DCs may contribute to elicit robust antitumor T cell immunity in the progression stage of tumor growth.

As a component of the adaptive immune system, cytotoxic CD8^+^ T cells play a vital role in anticancer immunity by releasing cytotoxic granules and killing tumor cells [39,40]. Notably, a recent study has shown that multifunctional CD8^+^ T cells that simultaneously express Th1 cytokines and/or CD107a as a marker for cytotoxic granules have a greater protective effect than monofunctional CD8^+^ T cells in a DC-based vaccine study against metastatic melanoma [41]. In addition, there are several studies in preclinical and clinical settings showing that a higher frequency of multifunctional CD8^+^ cells that produce IFN-γ, TNF-α, CD107a, and/or IL-2 have been observed in various models of cancer immunotherapy, such as T cell therapy, antibody therapy, and vaccine therapy [41,42,43,44,45]. Notably, CD4^+^ T cells are known to support survival and memory formation of tumor-specific multifunctional CD8^+^ T cells [46,47]. Furthermore, multifunctional CD4^+^ CTLs, which have characteristics and functions similar to those of cytotoxic CD8^+^ T cells, play an important role in the fight against cancer [28,39,48]. In this regard, inducing multifunctional CD4^+^ and CD8^+^ T cells is critical to the success of various therapeutic cancer strategies. Thus, we finally evaluated the multifunctional capacity of T cells induced by Ocs-P administration in tumor-bearing mice. Interestingly, Ocs-P administration showed higher levels of multifunctional CD4^+^ and CD8^+^ T cells capable of expressing multiple cytokines. Collectively, our results suggest that Ocs-P can promote the antitumor effect by generating multifunctional CD4^+^ and CD8^+^ T cells based on the restoration of tumor-associated DC maturation.

## 5. Conclusions

In summary, this study reports the immunological roles of Ocs-P in activation for DC maturation in vitro and the effect of Ocs-P as an immunostimulator for improvement of the anticancer immune response. The results revealed that Ocs-P promoted the expression of CD80, CD86, MHC-I, and MHC-II on DCs, along with the release of Th1-polarizing cytokines by activating MAPK and NF-ĸB signals. These Ocs-P-stimulated DCs activated the adaptive immune response by proliferation and differentiation of naive T cells into Th1 and activated cytotoxic CD8^+^ T cells. Notably, Ocs-P administration conferred strong anticancer T cell immunity (multifunctional CD4^+^ and CD8^+^ immune responses) and significant protection to mice that received colon carcinoma cells, suggesting that Ocs-P will be potentially useful in developing nutraceuticals to improve anticancer immunity. Nevertheless, the characterization and identification of novel immunogenic proteins contained in Ocs-P require further investigation. These additional studies may contribute to the development of effective immunological adjuvant targeted toward the treatment of infectious diseases (intracellular pathogens) and cancers.
